# A Rare Presentation of Imidacloprid Poisoning

**DOI:** 10.7759/cureus.35400

**Published:** 2023-02-24

**Authors:** Bhavya Chadalavada, Ritesh Baddam

**Affiliations:** 1 Internal Medicine, Gandhi Medical College, Hyderabad, IND

**Keywords:** environmental toxicology, imidacloprid, methylene blue, pesticide poisoning, medical toxicology

## Abstract

Acute poisoning with pesticides and insecticides can sometimes result in unexpected clinical manifestations. Awareness regarding all possible signs and symptoms of poisoning with these compounds can help in timely diagnosis and treatment. Deliberate ingestion of imidacloprid, a neonicotinoid insecticide, despite being minimally lethal to humans, proved to be life-threatening to a 14-year-old boy. A prompt diagnosis of methemoglobinemia followed by the administration of methylene blue led to successful recovery.

## Introduction

Acute poisoning with a wide range of pesticides and insecticides is witnessed commonly in our hospital. It is helpful to know the various clinical manifestations of each compound because most often the patient is unable to provide the name of the poison. Here, we report an unusual manifestation of a common poison.

## Case presentation

**Figure 1:**
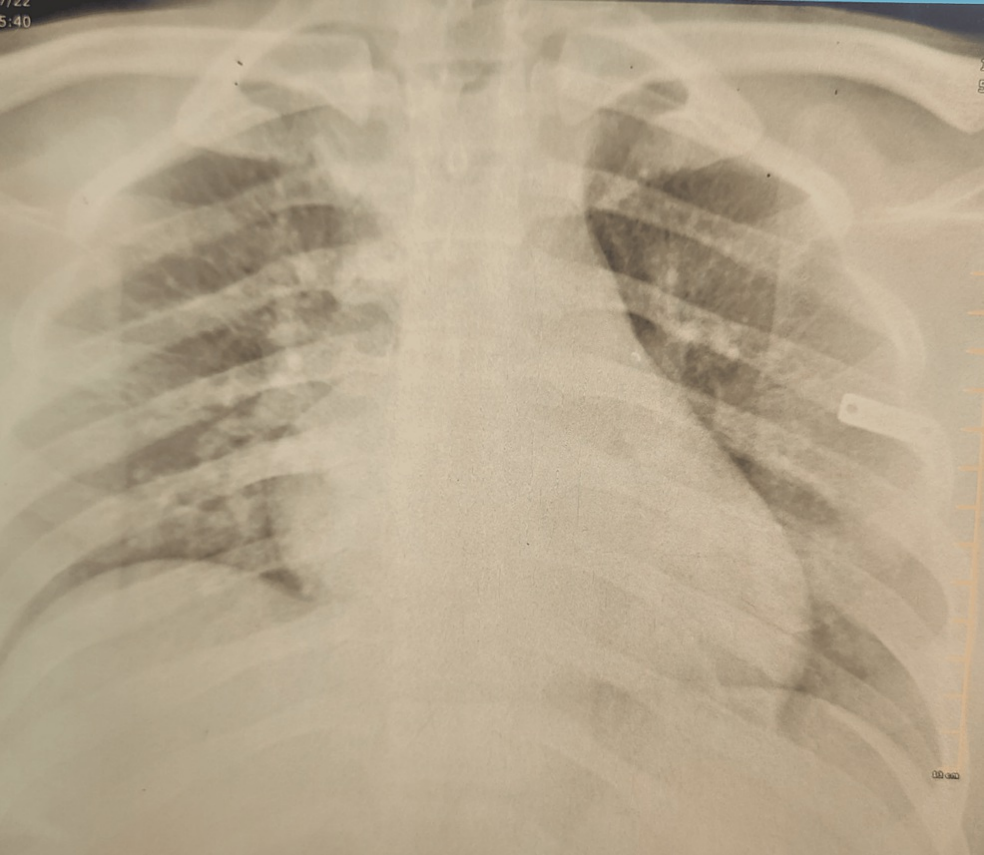
Chest X-ray anteroposterior view showing bilateral infiltrates.

A 14-year-old boy presented to the hospital after deliberate ingestion of 50 mL of imidacloprid mixed with alcohol. The patient was discovered by his attendees in an unconscious state approximately 30 minutes after the alleged intake. Upon arrival at the hospital, the patient was drowsy but arousable, dyspnoeic, and profusely sweating. History regarding the poison was taken and confirmed with the bottle that the attendees brought to the hospital, Confidor, imidacloprid 17.8% SL. On examination, the patient was drowsy, with a Glasgow Coma Scale (GCS) score of 8/15 E2V2M4, saturation of 78% at room air, respiratory rate of 30 breaths per minute, blood pressure of 120/80mmHg, and pulse rate of 60 beats per minute. His pupils were 3 mm, equal and reacting to light. There was no pallor, icterus, cyanosis, or pedal edema. Bilateral crepitations were heard on auscultation. Investigations showed leucocytosis. A chest X-ray showed bilateral infiltrates (Figure 1). An examination of serum electrolytes showed the following: sodium 145 mEq/L, potassium 3.6 mEq/L, and chloride 103 mEq/L. Renal function tests and liver function tests were normal.

The patient underwent gastric lavage. Despite high-flow oxygen, saturation levels remained between 70% and 80%. The patient was intubated and received antibiotics for suspected aspiration following alcohol intoxication. However, peripheral saturation levels did not improve, and his saturation on arterial blood gas (ABG) analysis was 98%. This saturation gap along with the dark color of his blood raised suspicion of methemoglobinemia. It was confirmed by meth-hemoglobin (met-Hb) on ABG which of 8% and met-Hb by spectrometry of 8.2%. Methylene blue 1 mg/kg was given as a bolus dose, and repeat ABG after one hour showed met-Hb of 4% and after 12 hours of 0.2%. Saturation improved and we were able to extubate him. A high dose of vitamin C was also administered daily. The patient’s sensorium improved and he was discharged after one week of hospital stay.

## Discussion

In the Asia-Pacific region, agrochemical poisoning is one of the primary causes of intentional suicide [1,2]. Organophosphates, organochlorines, and aluminum phosphide compounds are the most prevalent toxins and are the most common causes of mortality. They are an important aspect of the region’s agricultural landscape and are readily available at low prices. Imidacloprid is a neonicotinoid pesticide in the chloronicotinyl nitroguanidine chemical family that is not lethal to humans even at very high dosages [1,2]. Its International Union of Pure and Applied Chemistry name is 1-(6-chloro-3- pyridylmethyl)-N-nitroimidazolidin-2-ylideneamine and the Chemical Abstracts Service registry number is 138261-41-3. It acts as a competitive inhibitor at the nicotinic acetylcholine receptors interfering with the transmission of impulses leading to fatigue and paralysis. Central nervous system stimulation causes dizziness, drowsiness, disorientation, and coma. Autonomic nervous system stimulation causes sweating, dilated pupils, tachycardia, and hypertension [3].

In our case, the patient was initially treated for aspiration pneumonitis secondary to alcohol intoxication. Because imidacloprid has no known antidote, the immediate focus was on treating the presenting symptoms. If the hypoxia was secondary to pneumonia, low saturation levels on peripheral oximetry (SpO_2_) were expected to improve with oxygen, but, surprisingly, there was no change. Due to poor GCS and suspected respiratory depression, the patient was intubated and kept on mechanical ventilation, which did not show any improvement in his saturation levels. During the course of admission, saturation levels on ABG (SaO_2_) remained normal. This saturation gap led to the suspicion of methemoglobinemia. The met-Hb values were just 8.2%; patients typically begin to exhibit symptoms when levels exceed 10%. This may be explained by the fact that people with lung injury exhibit symptoms at lower concentrations of methemoglobin due to pre-existing reduced oxygen delivery.

Methemoglobinemia must be suspected when saturation on pulse oximetry is low but normal on ABG [3,4]. Methemoglobinemia is a condition in which the iron in hemoglobin is transformed from a ferrous (Fe^2+^) to ferric (Fe^3+^) state, resulting in impaired oxygen delivery to the tissues and causing brownish discoloration of blood [4,5]. Methylene blue is a heterocyclic aromatic compound that can reverse this transformation. It is given at a dose of 1-2 mg/kg (up to a total of 50 mg in adults) as a 1% solution in intravenous saline over three to five minutes [4,5]. Administration may be repeated at 1 mg/kg every 30 minutes to control symptoms.

## Conclusions

There is very little information regarding the toxicity of imidacloprid in humans, and whatever knowledge we have is gathered from a few published case reports and series. Such rare clinical manifestations of an easily available toxin question the need for testing blood levels of the toxin. This will make it simpler to investigate a the numerous clinical effects of the toxin and how each effect manifests at varying blood concentrations. This information is valuable for physicians, regulatory authorities, and the general public. If the harmful consequences of the poison in people are established along with the blood levels at which they occur, doctors will find it beneficial to recognize adverse effects such as methemoglobinemia and ensure timely treatment. Drugs and toxins are the most common cause of methemoglobinemia, and treatment with methylene blue can be life-saving. Further studies on the effects of insecticides on humans are required, and awareness programs regarding their toxicity must be implemented.
